# Limits of noise for autoregulated gene expression

**DOI:** 10.1007/s00285-018-1248-4

**Published:** 2018-05-24

**Authors:** Peter Czuppon, Peter Pfaffelhuber

**Affiliations:** 1 Abteilung fur Mathematische Stochastik, University of Freiburg, Eckerstr. 1, 79104 Freiburg, Germany; 2 Present Address: Department of Evolutionary Theory, Max-Planck Institute for Evolutionary Biology, Plön, Germany

**Keywords:** Intrinsic noise, Langevin approximation, Quasi-steady-state assumption, Chemical reaction network, Auto-regulated gene expression, 92C40, 60J60, 60F05

## Abstract

Gene expression is influenced by extrinsic noise (involving a fluctuating environment of cellular processes) and intrinsic noise (referring to fluctuations within a cell under constant environment). We study the standard model of gene expression including an (in-)active gene, mRNA and protein. Gene expression is regulated in the sense that the protein feeds back and either represses (negative feedback) or enhances (positive feedback) its production at the stage of transcription. While it is well-known that negative (positive) feedback reduces (increases) intrinsic noise, we give a precise result on the resulting fluctuations in protein numbers. The technique we use is an extension of the Langevin approximation and is an application of a central limit theorem under stochastic averaging for Markov jump processes (Kang et al. in Ann Appl Probab 24:721–759, 2014). We find that (under our scaling and in equilibrium), negative feedback leads to a reduction in the Fano factor of at most 2, while the noise under positive feedback is potentially unbounded. The fit with simulations is very good and improves on known approximations.

## Introduction

It is now widely accepted that gene expression is a stochastic process. The reason is that a single cell is a system with only one or two copies of each gene and of the order tens for mRNA molecules (Swain et al. 2002; Elowitz et al. 2002; Raj and van Oudenaarden 2008). Experimentally, this stochasticity can even be observed directly by single-cell measurements such as flow cytometry and fluorescence microscopy, which show the inherent fluctuations of protein numbers arising from cell to cell (Li and Xie 2011).

Usually, noise in gene expression is divided into an intrinsic and an extrinsic part (Swain et al. 2002; Raser and O’Shea 2005). While the intrinsic part leads to variation of protein numbers from cell to cell in the same environment, the extrinsic part is attributed to the different environmental conditions of the cell. In practice, ensemble averages eliminate intrinsic noise, while single-cell measurements over time can be thought of having a constant environment, thus eliminating extrinsic noise (Singh and Soltani 2013; Singh 2014).

Stochasticity in gene expression is not only interesting per se. Today, its role in evolution, development and cell fate decisions is under discussion (Kaern et al. 2005; Maamar et al. 2007; Fraser and Kærn 2009; Eldar and Elowitz 2010; Balázsi et al. 2011; Silva-Rocha and Lorenzo 2010; Wang and Zhang 2011). For instance, noisy gene expression can be detrimental for the survival of cells under harsh conditions (Mitosch et al. 2017; Fraser and Kærn 2009). Still, many cells have to function constantly. Therefore, mechanisms reducing and controlling the level of noise are beneficial for most of real systems.

Under the central dogma of molecular biology, modeling stochasticity of gene expression is straight-forward (see Paulsson 2005 for a review). A gene, which is either turned *on* or *off*, is transcribed into mRNA, which is translated into protein. Both, mRNA and protein are degraded at constant rates. Since the resulting chemical reaction network is linear, the master equation can be solved and all moments can be derived analytically. Most interestingly, the variance can be decomposed into the effects of switching the gene *on* and *off*, noise due to the finite life-time of mRNA, and random fluctuations in the production of protein (Paulsson 2005). It is often stated that gene expression tends to occur in bursts, which occur due to the short life-time of the *on*-state of the gene and due to the short life-time of mRNA (Kumar et al. 2015).

We are interested in the effect of self-regulation on gene expression noise. It is known that a negative feedback loop, i.e. a protein suppressing its own transcription (or translation) leads to a reduced noise, while positive feedback is attributed to increase noise (Lestas et al. 2010; Hornung and Barkai 2008). Although these findings are wide-spread, a complete mathematical analysis is lacking. At least, for negative feedback, Thattai and Oudenaarden (2001) and in more generality Swain (2004) quantify the effect of negative feedback using a linearization argument. The latter paper further analyzes different feedback models differing between translational and transcriptional autoregulation. Moreover, Dessalles et al. (2017) derive the equilibrium distribution using a multi-scale approach under negative feedback.

Most analyses of noise in unregulated gene expression rely on the master equation (e.g. Paulsson 2005). By the linearity of this equation, a solution can be given explicitly. Using the approximation that gene switching is so fast that it is effectively constantly transcribed to mRNA, this linearity can as well be used under negative feedback (Thattai and Oudenaarden 2001; Swain 2004). Our approach and also the one performed in Dessalles et al. (2017) differs in two ways. First, we are using martingale methods from stochastic analysis in order to describe the chemical system (Ethier and Kurtz 1986). Second, we can relax the assumption that the gene is transcribed effectively constantly, and therefore derive a more general result. Consequently, we are able to analyze noise in a truly non-linear system under a quasi-steady-state assumption.

The explicit expression we derive for the noise in the number of proteins is also the main difference between our findings and the results obtained in Dessalles et al. (2017). Since the authors of that paper are interested in the case of not very abundant proteins they compute a stationary distribution for the protein using martingale techniques in the context of birth–death processes as opposed to our stochastic diffusion setting.

While the full model of regulated gene expression (or any other chemical reaction network) is usually hard to study, considering an ODE approach instead, which approximates the full model, leads to new insights. Formally, a law of large numbers—usually referred to as a fluid limit—can be obtained connecting the stochastic and deterministic model (Kurtz 1970b; Darling 2002). While such a law of large numbers gives a deterministic limit, fluctuations are studied using central limit results; see Kurtz (1970a). The special situation for gene expression is that the gene and mRNA only have a few copies, while the protein is often in large abundance. Such multi-scale models are often studied under a quasi-steady-state assumption (Seegel and Slemrod 1989). Here, the species in low abundance are assumed to evolve fast, such that the slow, abundant, species only sense their time-average. For such a stochastic averaging, not only a law of large numbers is given e.g. by Ball et al. (2006), but also a central limit result has recently been obtained by Kang et al. (2014).

While a multi-scale approach to stochastic gene expression is not new (see Bokes et al. 2012; Dessalles et al. 2017), the analysis of fluctuations for such systems is not finished yet. In the case of multi-scale diffusion systems, Pardoux and Veretennikov (2001, 2003) derive a limit result for the slow components using a Poisson-equation. The results by Kang et al. (2014) are similar but are based on Markov jump processes instead of a diffusion limit framework. We apply the techniques of Kang et al. (2014) on the chemical reaction network of (un-)regulated gene expression. As our results show, fluctuations take into account all sources of noise and we give explicit formulas for the reduction of noise under negative feedback and the increase in noise under positive feedback.

## The model

We are dealing with the standard model of gene expression without and with feedback; see e.g. Dessalles et al. (2017). Using the terminology from Paulsson (2005), we write for the model without feedback (or the *neutral* model) 
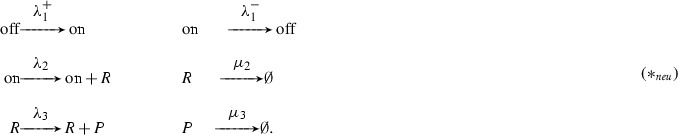
Here, *off* and *on* refer to an inactive and an active gene, respectively. The mRNA is given by *R*, and the protein by *P*. While the first line of chemical reactions models gene switching from *off* to *on* and back, the second line encodes transcription and degradation of mRNA, and the third line gives translation and degradation of proteins. Exchanging the first line by 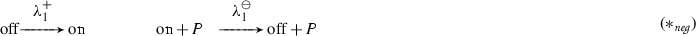
then models a negative feedback and 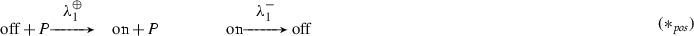
models a positive feedback. In all cases, we number the equations from left to right and from top to bottom by 1–6, so ${\mathcal {K}} = \{1,\ldots ,6\}$ is the set of chemical reactions. The species counts are given by $X_i$ for $i\in {\mathcal {S}}:=\{\text {off}, \text {on}, R, P\}$ for inactive and active gene, mRNA and protein, respectively. In the following we will scale the rates such that the gene switching and the mRNA production happens on a fast time-scale whereas the protein which is also present in higher abundances than the mRNA is evolving on a slower time-scale. This time-scale separation is frequently used in quantitative analyses of gene expression (see for instance Thattai and Oudenaarden 2001; Swain 2004; Ball et al. 2006; Bokes et al. 2012; Dessalles et al. 2017) since it allows to employ a quasi-steady-state assumption for the species evolving on the fast time-scale, cf. Kuehn (2015). It basically means that one first solves for the stationary points in the fast sub-system which are then used to describe the dynamics in the slow sub-system.

Thus, using some large constant *N*, we will make use of the following scaling for the abundances of chemical species

$$\begin{aligned} X_{\text {off}} = O(1), \quad X_{\text {on}} = O(1), \quad X_{R} = O(1), \quad X_{P} = O(N), \end{aligned}$$

or $X_i = O(N^{\alpha _i})$ for $i\in {\mathcal {S}}$ with

1 $$\begin{aligned}&\alpha _{\text {off}} = \alpha _{\text {on}} = \alpha _{R} = 0, \quad \alpha _{P} = 1. \end{aligned}$$

Reactions are scaled for all models such that we indeed get a time-scale separation, i.e. reaction constants are such that genes and mRNAs evolve much faster than protein numbers. Note, however, that due to $X_P = O(N)$, we need that the protein production rate needs to scale with *N*. We use the scaled rates $\kappa _2, \nu _2, \kappa _3, \nu _3 = O(1)$, which are given through

$$\begin{aligned} \lambda _2 = N \kappa _2, \quad \mu _2 = N \nu _2, \quad \lambda _3 = N\kappa _3, \quad \mu _3 = \nu _3. \end{aligned}$$

For the neutral model, we also set (with $\kappa _1^+, \kappa _1^- = O(1)$) 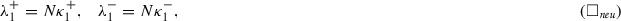
whereas for negative feedback (with $\kappa _1^+, \kappa _1^\ominus = O(1)$) 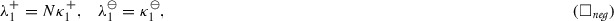
and for positive feedback (with $\kappa _1^\oplus , \kappa _1^- = O(1)$) 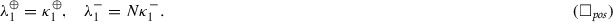
(Note that these scalings obey $\lambda _1^-, \lambda _1^\ominus X_P, \lambda _1^+, \lambda _1^\oplus X_P = O(N)$ which is necessary for the time-scale separation.) Setting (with $\alpha _i$ from (1))

2 $$\begin{aligned} V^N_i = N^{-\alpha _i} X_i, \quad i\in {\mathcal {S}} \end{aligned}$$

as the scaled number of genes, mRNA molecules and proteins, respectively, $V_i^N(0)$ the corresponding initial value and for *M* denoting the total copy number of genes, we have in the neutral case 
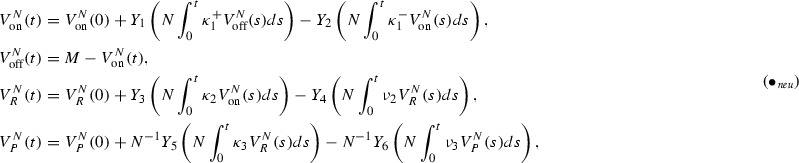
for independent, rate 1 Poisson processes $Y_1,\ldots ,Y_6$. (See (Anderson and Kurtz 2015) for a general introduction on theoretical chemical reaction networks.) The first equation changes in the case of negative feedback to 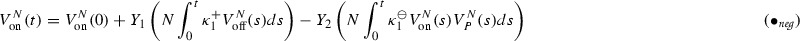
and in the case of positive feedback to 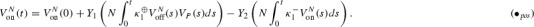
In the sequel, we will refer to the model without, with negative and positive feedback simply as ,  and , respectively. We understand all equations (), (), () as the bases for , equations (), (), () as the bases for  and all equations (), (), () as the bases for .

For a summary of the parameters and their scalings, see Table 1. We will refer to $\lambda$’s and $\mu$’s as the unscaled parameters and to $\kappa$’s and $\nu$’s as the scaled parameters.

**Table 1:** We use *N* as a scaling parameter throughout. Reaction rates either come in unscaled ($\lambda$’s and $\mu$’s) or in scaled ($\kappa$’s and $\nu$’s) versions

Parameter	Model	Meaning	Scaling
*N*		Scaling parameter	
$\lambda _1^+, \kappa _1^+$		Rate of switching genes on	$\lambda _1^+=\kappa _1^+ N$
$\lambda _1^\oplus , \kappa _1^\oplus$			$\lambda _1^\oplus = \kappa _1^\oplus$
$\lambda _1^-, \kappa _1^-$		Rate of switching genes off	$\lambda _1^-=\kappa _1^- N$
$\lambda _1^\ominus , \kappa _1^\ominus$			$\lambda _1^\ominus = \kappa _1^\ominus$
$\lambda _2, \kappa _2$		Rate of mRNA production	$\lambda _2 = \kappa _2 N$
$\mu _2, \nu _2$		Rate of mRNA degradation	$\mu _2 = \nu _2 N$
$\lambda _3, \kappa _3$		Rate of protein production	$\lambda _3 = \kappa _3 N$
$\mu _3, \nu _3$		Rate of protein degradation	$\mu _3 = \nu _3$
*M*		Total number of genes	$M=O(1)$

## Results

The following results are all stated in terms of the scaled parameters ($\kappa$’s and $\nu$’s and $V_P^N$). For the corresponding formulas using unscaled parameter notation, see Appendix E.

### A limiting process for the amount of protein

The following result can be obtained using a quasi-steady-state assumption. It relies on the method of stochastic averaging; see e.g. Ball et al. (2006). Basically, it says that, using the law of large numbers for Poisson processes, i.e. $Y(t) \approx t$ for large *t*, and the scaled parameter set we find

3 $$\begin{aligned} V_P^N(t) \approx V_P^N(0) + \int _0^t \left( \kappa _3 {\mathbb {E}}_\pi \left[ V_R^N(s)\right] - \nu _3 V_P^N(s) \right) ds, \end{aligned}$$

where ${\mathbb {E}}_\pi [\cdot ]$ denotes expectation with respect to the equilibrium dynamics of the fast species $V^N_{\text {off}}, V^N_{\text {on}}$ and $V_R^N$ for a fixed amount of protein, $V_P^N$. Note that we use $\Rightarrow$ for weak convergence of stochastic processes (Ethier and Kurtz 1986).

#### Theorem 1

(Law of Large Numbers) We consider the models ,  and  and assume that the initial condition converges in distribution, i.e. . Then, , where $v_P$ solves

$$\begin{aligned} {\dot{v}}_P = F(v_P), \end{aligned}$$

with4In particular, the equilibrium, i.e. $F(v_P^*)=0$, is given by5


#### Proof

We apply results from Ball et al. (2006) and only sketch the proof. In order to derive Eq. (4) we need to replace $V_{\text {on}}$ and $V_R$ (the fast variables) in the equation for $V_P^N$ by their equilibria assuming that $V_P^N$ is constant. Computing these equilibria is done using the corresponding lines in (), () and (). The resulting distribution $\pi$ then is the equilibrium on the fast time-scale. For $v_P$ fixed they readandPlugging this equilibrium into (3) which is the limit for large *N* of the corresponding equation in (), we obtain that

6 $$\begin{aligned} {\dot{v}}_P&= \kappa _3 {\mathbb {E}}_\pi [V_R] - \nu _3 v_P = F(v_P) \end{aligned}$$

with *F* as in (4). Computation of the equilibria is standard by solving $F(v_P)=0$. In particular, we have to solve

$$\begin{aligned} -M\kappa _1^+ \kappa _2\kappa _3 + \kappa _1^+\nu _2\nu _3 v_P + \kappa _1^\ominus \nu _2\nu _3 v_P^2&=0 \quad \text { or }\quad v_P^2 + \frac{\kappa _1^+}{\kappa _1^\ominus } v_P - \frac{M\kappa _1^+ \kappa _2\kappa _3}{ \kappa _1^\ominus \nu _2\nu _3} =0 \end{aligned}$$

for the equilibrium of . $\square$

### Approximate variance and Fano factor for the amount of protein

Our goal is to derive the variance in protein numbers under ,  and . While  is solved explicitly elsewhere, e.g. in Paulsson (2005), some approximations have to be made for  and . One idea might be to use a Langevin approximation and write

$$\begin{aligned} V_P^N(t)&\approx V_P^N(0) + \int _0^t \left( \kappa _3 V_R^N(s) - \nu _3 V_P^N(s) \right) ds \\&\qquad \qquad + \frac{1}{\sqrt{N}}\int _0^t\sqrt{\kappa _3 V_R^N(s) + \nu _3 V_P^N(s)}dW_s \\&\approx V_P^N(0) + \int _0^t F\left( V_P^N(s)\right) ds + \frac{1}{\sqrt{N}}\int _0^t \sqrt{b\left( V_P^N(s)\right) }dW_s \end{aligned}$$

withComparing $V_P^N$ and $v_P$, where $v_P$ is the exact solution of ${\dot{v}}_P = F(v_P) = \kappa _3 {\mathbb {E}}_\pi [V_R] - \nu _3 v_P$ with *F* from Theorem 1, we assume that $V_P^N \approx v_P + \frac{1}{\sqrt{N}}U$ for some stochastic process *U*. The random process *U* will then account for the fluctuations which are not captured by the deterministic approximation above. Hence,

$$\begin{aligned} \frac{1}{\sqrt{N}}dU&= dV_P^N - dv_P \approx \left( F\left( V_P^N\right) - F(v_P)\right) dt + \frac{1}{\sqrt{N}}\sqrt{b\left( V_P^N\right) }dW \\&\approx F'(v_P)\left( V_P^N - v_P\right) dt + \frac{1}{\sqrt{N}}\sqrt{b\left( V_P^N\right) }dW\\&\approx \frac{1}{\sqrt{N}} \left( F'(v_P) U dt + \sqrt{b\left( V_P^N\right) }dW\right) \end{aligned}$$

and therefore

7 $$\begin{aligned} \begin{aligned} dV_P^N&= dv_P + \frac{1}{\sqrt{N}} dU \approx \left( F(v_P) + \frac{1}{\sqrt{N}} F'(v_P)U\right) dt + \frac{1}{\sqrt{N}} \sqrt{b\left( V_P^N\right) } dW. \end{aligned} \end{aligned}$$

This approach builds on applying a quasi-steady-state assumption whenever possible, i.e. when averaging over the on/off-state of genes in order to derive the deterministic dynamics of $v_P$ using *F*, and the number of mRNA, which is approximated by its mean in order to derive *b*. Consequently, fluctuations arising from these two mechanisms cannot be accounted for in the resulting variance. As a result, fluctuations read off from (7) will be too small.

In contrast, as an application of Kang et al. (2014) (see also Appendix A), we derive the following central limit result, which takes into account all fluctuations in leading order. Precisely, our next goal is to show that $\sqrt{N}(V_P^N - v_P)$ converges and to determine the limiting process. This limit will then provide the error due to noise between the deterministic approximation $v_P$ and the stochastic process $V_P^N$ of order $\sqrt{N}$. In the proof, we will make use of the method developed by Kang et al. (2014).

#### Theorem 2

(Central Limit Theorem) Let $V_P^N, v_P$ and *F* be as in Theorem 1 and assume further weak convergence of the initial conditions:Then, for the models  and , , where *U* solves

8 $$\begin{aligned} U(t) = U(0) + \int _0^t \sqrt{c(v_P(s))}dW(s) + \int _0^t F'(v_P(s))U(s) ds, \end{aligned}$$

with *W* the one-dimensional standard Brownian motion and9


Hence, we see that, in contrast to the Langevin approach (7) above, fluctuations arising from gene switching and RNA dynamics are also accounted for in Theorem 2; see also Sect. 3.3 for an interpretation of the individual terms. For the difference between the Langevin approximation and our result and its implications see also Sect. 4.3.

The proof of the Theorem is given in Appendix B. Briefly, we apply the stochastic averaging principle on multiple time scales developed in Kang et al. (2014). The whole approach is revisited in Appendix A. There we also state the conditions which need to be satisfied for the theory to apply. Amongst others these include solving a certain Poisson equation which enables a clean time-scale separation.

#### Remark 1

(*Deriving the Fano factor in equilibrium*) While (8) provides a dynamical result along paths of $X_P$, we can also use this approximation and study the process in equilibrium by setting $X_P(0)=v_P^*N$, where $v_P^*$ is the unique solution of $F(v_P)=0$ given by (5).

In order to compute the approximate variance of $V_P^N$, when started in the equilibrium $v_P^*$, we make use of the fact that the stochastic differential equation (SDE) in (8) is solved by an Ornstein–Uhlenbeck process. In particular, we obtain at late times, i.e. when the process reached its equilibrium (recall that $X_P^N = NV_P^N$ is the total number of proteins)

10 $$\begin{aligned} \frac{{\mathbb {V}}[X_P]}{{\mathbb {E}}[X_P]} \approx \frac{N{\mathbb {V}}[V_P^N]}{{\mathbb {E}}[V_P^N]} \approx \frac{{\mathbb {V}}[U]}{v_P^*} \approx - \frac{c(v_P^*)}{2F'(v_P^*)v_P^*} \end{aligned}$$

with $F(\cdot )$ from (4), $v_P^*$ from (5) and $c(\cdot )$ from (9). Since no factor *N* appears on the right hand side, some authors call the Fano factor dimensionless. Empirically, it was found e.g. by Bar-Even et al. (2006), that for all classes of genes and under all conditions, the variance in protein numbers was approximately proportional to the mean, which is again reminiscent of the lacking *N* in the Fano factor above.

We note here that this approach of computing the Fano factor of $X_P^N$ in equilibrium was achieved by an unjustified exchange of limits. Namely, for the approximate Fano factor of $X_P^N$ in equilibrium, we would have to perform $t\rightarrow \infty$ first, and only then compute $N\rightarrow \infty$, but our approach exchanged this limit.

In order to compute the right hand side of (10), note that11Plugging in the equilibrium $v_P^*$ from (5) for  and , we obtain in particular thatand therefore12In addition,13Hence, plugging these quantities into (10) gives (with $X_P^*= N v_P^*$; see also (42) for the Fano factor in terms of unscaled parameters)14


### Interpretation of the Fano factor

The expressions above in Eq. (14) are not only a result from strict calculations but can also be interpreted in biological terms. For instance, for negative feedback, we find—as in the neutral case—contributions from randomness in gene switching, translation and transcription. Moreover, the negative feedback pushes the amount of protein faster back to its equilibrium value for a burst of gene expression. This results in the denominator in15which has the biggest noise-reducing effect of negative feedback which we will study hereafter.

Adjusted explanations hold in the cases of no or positive feedback.

### Comparing the noise in ,  and 

It is frequently reported that a negative feedback in gene expression results in a reduced variance (noise) of protein levels, whereas a positive feedback enhances noise (Lestas et al. 2010; Hornung and Barkai 2008). These observations can be made precise by our results from above. Here, we report some consequences on the equilibrium variance and the Fano factor, ${\mathbb {V}}[X_P]/{\mathbb {E}}[X_P]$.

**Fig. 1:**
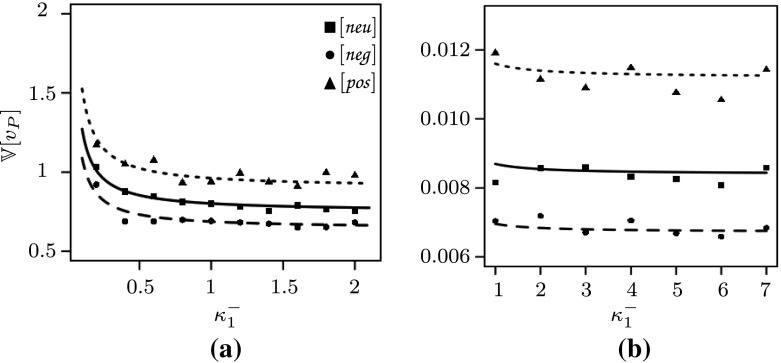
Simulations and theoretical results with a fixed mean of proteins, **a** $N v_P^*= 1250$, **b** $N v_P^*= 60$. The gene association and dissociation rates are varied, i.e. in (**a**) $\kappa _1^-=(0.2,0.4,\dots ,2)$ and in (**b**) $\kappa _1^- = (1,2,\dots ,7)$. The gene association rate $\kappa _1^+$ is then chosen such that the protein mean equals 1250 or 60 in each case, respectively. Furthermore, these rates are adjusted in the cases of negative and positive feedback according to $\kappa _1^\ominus := \kappa _1^-/ v_P^*$ and $\kappa _1^\oplus := \kappa _1^+ / v_P^*$, respectively. The other parameters are given by $M=1, N=100, \kappa _2=3, \nu _2=1, \kappa _3=5$ and $\nu _3=1$ in (**a**). For figure (**b**) we chose parameters as in (Anderson and Kurtz 2015, Figure 2.1), i.e. $M=1,N=100,\kappa _2 = 2, \nu _2 = 0.25,\kappa _3 =0.1, \nu _3 = 1$. The solid, dotted, dashed lines are the theoretical predictions in the no, positive and negative feedback cases, respectively. Each data point is derived from 1000 Monte Carlo simulations (cf. Gillespie 1977) of the full system given by  and

For a fair comparison, we use the models ,  and  for equal values of $v_P^*$. Consider a model  with parameters $\kappa _1^+, \kappa _1^-, \kappa _2, \kappa _3, \nu _2, \nu _3$ and let $v_P^*$ be the equilibrium from (5). In addition, consider a model  with $\kappa _1^\ominus := \kappa _1^-/v_P^*$ and all other parameters as above and a model  with $\kappa _1^\oplus := \kappa _1^+/v_p^*$ and all other parameters as above. Then, from (4), we see that all models have $v_P^*$ as their unique deterministic limit with the same $c(v_P^*)$ from (13). Setting  and  as the variance of the model without, with negative and with positive feedback, respectively, and plugging in all quantities in (14) then gives (note that the mean cancels out; see (43) for a version with unscaled parameters)16In particular, we see that the variance is reduced in  and increased in , as expected; see also Fig. 1. Moreover, the graphs show that the performed simulations fit our predictions well for both higher (a) and lower (b) values of $v_P^*$.

Additionally, we see from Eq. (16) that the change in noise is maximal if the gene is *off* most of the time, while still having the same amount of protein as in the unregulated (neutral) case. This finding is reminiscent of the fact that gene expression comes in bursts. The burstiness is most extreme if the gene is *on* only for a short time, producing a large amount of mRNA, and afterwards *off* for a long period. Especially, we see that for $\kappa _1^\ominus \rightarrow \infty$ the maximal reduction due to negative feedback is twofold while the increase in noise is unbounded for $\kappa _1^- \rightarrow \infty$ in case of positive auto-regulation of the protein.

### Negative feedback in a simpler model

In the literature simpler models of gene expression are studied as well. Here, only two molecular species are involved. Either, the gene is constitutively expressed, therefore ignoring the state of the gene, or translation is neglected and the gene is assumed to be transcribed leading to protein in one step (Hornos et al. 2005; Ramos et al. 2015, 2011; Shahrezaei and Swain 2008). In either case, we have the model similar tofor , whereas for  and , we take () and () instead of the first line, respectively. We note that this model arises from the full model described in (), () and (), when letting $\nu _2 = \kappa _2 \rightarrow \infty$. Hence, we obtain the following approximation for the simpler model17When comparing these equations with (15) we see that all the terms containing explicit mRNA noise disappeared. Thus, we still have contributions from gene activation and protein production and degradation as well as the scaling factor due to the auto-regulation.

### Refining the Fano factor

Here, we again consider the original model given by equations (), () and (), but use a different scaling for the mRNA. We assume that not only the protein evolves on the slow time-scale but also the mRNA production and degradation. This is a slightly more complex model since we cannot average the number of mRNA molecules when analyzing the protein fluctuations. This model allows us to compare our results in a more straightforward way with results obtained previously in Thattai and Oudenaarden (2001), Swain (2004); see Sects. 4.2 and 4.3.

**Fig. 2:**
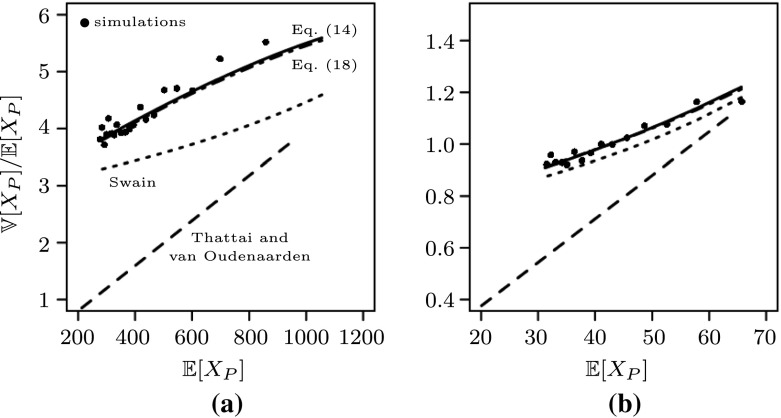
Simulations and theoretical results of gene expression with negative feedback. The mean given on the x-axis is varied and plotted against the Fano factor on the y-axis. The solid line represents (14), the dash-dotted line the result in (18), the dashed line the result from Thattai and Oudenaarden (2001) given in (22) and the dotted line the Fano factor calculated in Swain (2004) given in (23). The parameters for the simulations are chosen as follows: **a** $M=1, N=100, \lambda _1^+=250, \lambda _1^{\ominus }= (0.2,0.4,\dots ,4), \lambda _2=300,\mu _2=100, \lambda _3=500,\mu _3=1$; **b** $M=1,N=100,\lambda _1^+=300, \lambda _1^{\ominus }=(1,2,\dots ,15), \lambda _2=200,\mu _2=25,\lambda _3=10,\mu _3=1$. The bullets represent the estimated Fano factors of the full system  obtained from 1000 Monte Carlo simulations (cf. Gillespie 1977) in (**a**) and 5000 in (**b**) for each value of $\lambda _1^\ominus$

In order to account for the new scaling we set $\mu _2 = O(1)$ and $\lambda _3 = O(1)$. As scaled variables, we introduce ${{\widetilde{\nu }}}_2 = \mu _2$ and ${\widetilde{\kappa }}_3 = \lambda _3$. In this model, we have $X_R = O(N)$, but still $X_P=O(N)$. With these assumptions it is possible to derive a refined formula for the Fano factor for $X_P$ in equilibrium. Precisely, we compute in Appendix D—see Eqs. (37), (39) and (40)18withNote that this equation approximately gives (14) for ${\widetilde{\nu }}_2, {\widetilde{\kappa }}_3\gg 1$. However, adding interpretations as in Sect. 3.3 is not straight-forward since the additional terms in (18) stem from interactions between RNA and protein dynamics. In practice (and in our simulations below), the life-time of proteins is much larger than the life-time of mRNA, such that (18) does not produce a better fit than (14); see also Fig. 2 and compare the dash-dotted and the solid line. Therefore, when not stated otherwise, we will use (14) in the sequel.

## Comparison to previous results

Here, we compare our results in the neutral case with those obtained in Paulsson (2005), and in the case of negative feedback with the formulas for the Fano factor derived in Dessalles et al. (2017), Ramos et al. (2015), Swain (2004) and Thattai and Oudenaarden (2001).

### The neutral case, Paulsson (2005)

In Paulsson (2005) the neutral model  was studied without assuming any scalings of the parameter $\lambda _i,\mu _i$ or of the number of mRNA molecules or proteins. Setting

$$\begin{aligned} \tau _1 = \frac{1}{\lambda _1^- + \lambda _1^+}, \quad \tau _2 = \frac{1}{\mu _2}, \quad \tau _3 = \frac{1}{\mu _3}, \end{aligned}$$

as the expected life-times of a change in gene activity, mRNA and protein, respectively, we see that the Fano factor in equilibrium obeys (see equation (4) in Paulsson (2005))

$$\begin{aligned} \frac{{\mathbb {V}}[X_P]}{{\mathbb {E}}[X_P]}&= 1 + \frac{\lambda _3}{\mu _3}\frac{\tau _2}{\tau _2 + \tau _3} + \frac{{\mathbb {E}}[X_P] \lambda _1^-}{M\lambda _1^+} \frac{\tau _1}{(\tau _2 + \tau _3)(\tau _1 + \tau _3)} \frac{\tau _1\tau _2 + \tau _2\tau _3 + \tau _1\tau _3}{\tau _1 + \tau _2} \\&{\mathop {\approx }\limits ^{\tau _3\gg \tau _1, \tau _2}}1 + \frac{\lambda _3}{\mu _2} + \frac{\lambda _1^-\lambda _2\lambda _3}{\mu _2\mu _3(\lambda _1^- + \lambda _1^+)} \frac{\tau _1}{\tau _3} = 1 + \frac{\lambda _3}{\mu _2} + \frac{\lambda _1^-\lambda _2\lambda _3}{\mu _2(\lambda _1^- + \lambda _1^+)^2}. \end{aligned}$$

The approximation in the last line corresponds to our scaling, which is exactly such that $\tau _3 \gg \tau _1, \tau _2$ (since $\mu _3 \ll \mu _1, \mu _2$), i.e. the protein is much more stable than mRNA and the state of the gene. Thus, our approximation of the Fano factor in Eq. (14) (or rather its version in unscaled parameters in Eq. (42)) is in line with (Paulsson 2005).

### Negative feedback, Thattai and Oudenaarden (2001)

In Thattai and Oudenaarden (2001), a linearization of  was studied in the case of fast on- and off-switching of the gene. In particular, this will mean that both, $\kappa _1^-, \kappa _1^+ \gg 1$. In the following, we derive their result within our framework. Therefore, consider as in the proof of Theorem 1 that

$$\begin{aligned} {\mathbb {E}}_\pi [V_{\text {on}}] = \frac{M\kappa _1^+}{\kappa _1^\ominus v_P + \kappa _1^+}. \end{aligned}$$

Then, averaging out the gene state, we obtain the following system (compare with ())

19 $$\begin{aligned} V^N_R(t)&= V^N_R(0) + Y_3\left( N \int _0^t \kappa _2 \frac{M\kappa _1^+}{\kappa _1^\ominus V^N_P(s) + \kappa _1^+} ds\right) - Y_4\left( N \int _0^t \nu _2 V^N_R(s)ds\right) ,\nonumber \\ V^N_P(t)&= V^N_P(0) + N^{-1} Y_5\left( N \int _0^t \kappa _3 V^N_R(s)ds\right) - N^{-1} Y_6\left( N \int _0^t \nu _3 V^N_P(s)ds\right) . \end{aligned}$$

Assuming, like in Thattai and Oudenaarden (2001), that the equilibrium effective mRNA-production is a linear function, i.e. letting

20 $$\begin{aligned} \kappa _1^\ominus v_P^*\ll \kappa _1^+, \end{aligned}$$

such that we basically model a constitutively expressing gene, we can further approximate $V_R^N$ by

21 $$\begin{aligned} V^N_R(t)&= V^N_R(0) + Y_3\left( N \int _0^t M\kappa _2\left( 1 - \frac{\kappa _1^\ominus }{\kappa _1^+} V^N_P(s)\right) ds\right) - Y_4\left( N \int _0^t \nu _2 V^N_R(s)ds\right) . \end{aligned}$$

Now, the system (19) and (21) is exactly as on p. 3 in Thattai and Oudenaarden (2001) with

$$\begin{aligned} k_0 = NM\kappa _2, \quad k_1 = \frac{M\kappa _1^\ominus \kappa _2}{\kappa _1^+}, \quad k_P = N\kappa _3, \quad \gamma _R = N\nu _2, \quad \gamma _P = \nu _3. \end{aligned}$$

Plugging these variables into equation (3) of Thattai and Oudenaarden (2001) we obtain in equilibrium with

$$\begin{aligned} \eta = \frac{\gamma _P}{\gamma _R} = \frac{\nu _3}{N\nu _2}, \quad b = \frac{k_P}{\gamma _R} = \frac{\kappa _3}{\nu _2}, \quad \phi = \frac{k_1}{\gamma _P} = \frac{M\kappa _1^\ominus \kappa _2}{\kappa _1^+\nu _3} \end{aligned}$$

that (note that $\eta$ is negligible since *N* is large and $\phi$ is small by (8))

22 $$\begin{aligned} \frac{1}{N} {\mathbb {E}}[X_P]&= \frac{1}{N} \left( \frac{1}{1+b\phi }\right) \frac{k_0 b}{\gamma _P} \approx \left( 1 - \frac{M\kappa _1^\ominus \kappa _2\kappa _3}{\kappa _1^+\nu _2\nu _3}\right) \frac{M\kappa _2\kappa _3}{\nu _2\nu _3} \nonumber \\ \frac{{\mathbb {V}}[X_P]}{{\mathbb {E}}[X_P]}&= \left( \frac{1-\phi }{1+b\phi } \cdot \frac{b}{1+\eta } +1\right) \approx 1 + b(1-\phi )(1-b\phi )\nonumber \\&\approx (1+b)(1-b\phi ) = \left( 1 + \frac{\kappa _3}{\nu _2}\right) \left( 1 - M\frac{\kappa _2\kappa _3}{\nu _2\nu _3} \frac{\kappa _1^\ominus }{\kappa _1^+}\right) \nonumber \\&\approx \left( 1 + \frac{\kappa _3}{\nu _2}\right) \left( 1 - \frac{\kappa _1^\ominus }{\kappa _1^+} v_P^*\right) . \end{aligned}$$

Considering our scaling assumption, and further assuming (20), Eq. (14) can be simplified to

$$\begin{aligned} \begin{aligned} \frac{{\mathbb {V}}[X_P]}{{\mathbb {E}}[X_P]}&\approx \left( \frac{\kappa _1^\ominus v_P^*\kappa _2\kappa _3}{(\kappa _1^+ + \kappa _1^\ominus v_P^*)^2 \nu _2} + \frac{\kappa _3}{\nu _2} + 1\right) \left( 1 + \frac{\kappa _1^\ominus v_P^*}{\kappa _1^\ominus v_P^*+ \kappa _1^+}\right) ^{-1} \nonumber \\&\approx \left( 1+\frac{\kappa _3}{\nu _2}\right) \left( 1-\frac{\kappa _1^\ominus v_P^*}{\kappa _1^+}\right) \end{aligned} \end{aligned}$$

which equals the expression from (22). The results of this approximation are compared to our result in Fig. 2.

Recalling our exact result for the Fano factor from Eq. (14), we note that due to the linearization of the mRNA expression and thus a basically constant mRNA production, the noise emerging from the random gene switches is not adequately represented in the formula obtained in Thattai and Oudenaarden (2001). To be more precise, in contrast to our formula in (14), the effect of mRNA noise due to gene switching (first term in first bracket) is not taken into account at all. Additionally, the negative feedback (last bracket in (22)) does not affect the noise in the same way as it does in the exact formula (14). As can be seen in Fig. 2, when comparing the solid and the dashed lines, these effects lead to an underestimation of the actual noise which is produced by an exact simulation of .

### Negative feedback, Swain (2004)

As explained around (7), the usual Langevin approximation cannot account for all fluctuations when a quasi-steady-state assumption is made. (Precisely, it cannot account for fluctuations in the averaged variables.) In Swain (2004), a Langevin approach is carried out in order to analyze fluctuations in autoregulatory gene expression in the cases of transcriptional and translational feedback. The author considers the mRNA and the protein to evolve on the same time-scale whereas the gene (or DNA) is considered to be on a faster time-scale, see also Sect. 3.6. For transcriptional feedback (which we study here), Swain obtains in his equation (5)—see below for the transformation of his results into our parameters (unscaled, and scaled)23with . This corresponds exactly to (18)—see also Eq. (44) in Appendix E for the unscaled version–with a missing term in the numerator, namely . This term arises from fluctuations in gene activation, which was averaged out in calculations done in Swain (2004). At least, (23) arises from (18) if we assume that $\kappa _1^\ominus v_P^*, \kappa _1^+ \gg 1$, i.e. fast gene switching.

For a comparison of the two results we refer to the solid and dotted lines in Fig. 2 where we see that due to the missing term in the approximation obtained by Swain, his result slightly underestimates the Fano factor resulting from simulations of . However, the difference becomes smaller for a lower number of expected proteins, see Fig. 2b. This can be explained by the difference between Eqs. (23) and (18). The missing term in Swain’s derivation can be related to noise emerging from the gene switching. These processes however are given by the overall parameter configuration. Hence the larger difference between the solid and dotted lines in (a) when compared to (b) simply emerges from the corresponding term in (a) being larger than in (b). Thus, it therefore has a stronger effect on the overall fluctuations in protein numbers.

In order to obtain Eq. (23), reference (Swain 2004) uses $d_0={\widetilde{\nu }}_2, d_1=\nu _3, v_1={\widetilde{\kappa }}_3, \langle M\rangle = \frac{\kappa _1^+\kappa _2}{(\kappa _1^\ominus v_P^*){\widetilde{\nu }}_2}$ and

$$\begin{aligned} \epsilon _c = \frac{2}{1+\sqrt{1+4\frac{\kappa _1^\ominus \kappa _2{\widetilde{\kappa }}_3}{\kappa _1^+ {\widetilde{\nu }}_2\nu _3}}} \end{aligned}$$

for the Fano factor for the auto-regulatory gene expression with negative (transcriptional) feedback. Expressing terms as in Sect. 3.6, we rearrange his equation (5) (recall (5) and note that $v_P^*/ {\mathbb {E}}_\pi [V_R] = {\widetilde{\kappa }}_3/\nu _3$; see (6)) with $M=1$
showing (23).

### Negative feedback, Dessalles et al. (2017)

Our result for  has Theorem 2 of Dessalles et al. (2017) as a limit result. They study a similar model (with $M=1$), but with a scaling such that $\lambda _3, \mu _3 = O(1)$ and $\lambda _1^\ominus = O(N)$, leading to low (i.e. *O*(1)) abundance of protein in the system. Assuming the same time-scale separation as in our model, i.e. gene switching and mRNA processes evolve on a fast time-scale, the resulting birth–death process for *P* has a stationary distribution which they compute explicitly. Moreover, setting

$$\begin{aligned} \rho = \frac{\lambda _1^+\lambda _2 \lambda _3}{\lambda _1^\ominus \mu _2\mu _3}, \end{aligned}$$

they obtain in their Corollary 4.1 that, in equilibrium,

24 $$\begin{aligned} \frac{{\mathbb {V}}[X_P]}{{\mathbb {E}}[X_P]} {\mathop {\approx }\limits ^{\rho \rightarrow \infty }}\frac{1}{2}. \end{aligned}$$

For large $\rho$, our model simplifies to $v_P^*\approx \sqrt{\rho }$, and then (14) gives the same limit.

### Negative feedback in a simpler model, Ramos et al. (2015)

In Ramos et al. (2015), the authors derive in their equation (11) the Fano factor for the simplified model, which we introduced in Sect. 3.5. (We note that their model slightly differs since the protein binds to the gene and therefore cannot degrade in this state.) Their results connect the Fano factor to the covariance of the number of active genes and the number of proteins in equilibrium. Since they also give the limiting distribution (in terms of a confluent hypergeometric function), they can evaluate this covariance and also the Fano factor numerically. From their Figure 2, one can see that—if proteins are somewhat abundant—the Fano factor stabilizes around 1/2 for various parameter combinations; a result reminiscent of Dessalles et al. (2017) as discussed above.

### Negative feedback for small amounts of protein

Theorems 1 and 2—and all subsequent calculations—only hold under the scaling described in $\Box _*$ or in Appendix D. In these scalings, we have that $X_P = O(N)$, i.e. there the protein is abundant. In this case, we see from (14) that the Fano factor is at least 1/2. In Sect. 4.4, we discussed the results by Dessalles et al. (2017), where $X_P = O(1)$ is used, but the limiting result for $\rho \rightarrow \infty$ implies that proteins are abundant and leads to a Fano factor of at least 1/2.

However, the scaling of Dessalles et al. (2017) also allows for smaller values for the Fano factor, which is called the infra-Fano regime in Ramos et al. (2015). Precisely, consider the model from Dessalles et al. (2017), where the scaling

$$\begin{aligned} \lambda _1^+, \lambda _1^\ominus , \lambda _2, \mu _2 = O(N), \quad \lambda _3, \mu _3 = O(1) \end{aligned}$$

is used. It is shown that $X_P$ converges towards a birth–death process with

$$\begin{aligned} \text {death rates }\delta _n := \mu _3 n \text { and birth rates } \beta _n := \frac{\lambda _3}{\mu _2} \frac{\lambda _2 \lambda _1^+}{\lambda _1^+ + \lambda _1^\ominus n}, \end{aligned}$$

if there are *n* proteins. For this process, they compute the equilibrium distribution

$$\begin{aligned} \pi (n) = \frac{1}{Z} \prod _{i=0}^{n-1} \frac{\rho }{(i+1)(i + \lambda _1^+/\lambda _1^\ominus )} \end{aligned}$$

with $\rho = \lambda ^+_1 \lambda _2 \lambda _3 / (\lambda _1^\ominus \mu _2 \mu _3)$ and *Z* as a normalizing constant. In this case, one can see that for $\lambda _1^\ominus \gg \lambda _1^+$, $\pi$ is concentrated around 1, i.e. there is a single molecule of the protein and hence, the Fano factor becomes arbitrarily small. Since this in particular means a Fano factor below 1/2, Ramos et al. (2015) call this the infra-Fano regime.

## Conclusion

Quantifying noise in gene expression is essential for understanding regulatory networks in cells (Thattai and Oudenaarden 2001). Our results capture most of the previously derived results. While negative feedback is known to reduce noise under auto-regulated gene expression, we improve on the quantification of this effect, i.e. our results account for all possible sources of noise due to gene activation, mRNA fluctuations and the protein processes itself. We note that our results require that proteins are abundant. Since the infra-Fano regime described in Sect. 4.6 relies on small amounts of protein, our results do not recover this regime.

In addition, we provide the same quantification of noise also for positive feedback, where noise is increased. In particular, (14) shows that the average time the gene is *off* determines the reduction of noise in all cases relative to unregulated genes; see also Grönlund et al. (2013). As we saw earlier, for both, negative and positive feedback, noise difference between the non-regulated and the model with feedback is largest if the gene is *off* most of the time. This can be interpreted by the burstiness of gene expression. It is largest for genes which are *off* for long times and then turned *on* for a short time in which mRNA is produced. Interestingly, previous approaches mostly gave approximations for noise for negative feedback if switching the gene on and off is very fast (Thattai and Oudenaarden 2001; Swain 2004) and if the gene is *on* most of the time (Thattai and Oudenaarden 2001) or in a simplified model (Ramos et al. 2015). Hence, all previous papers could not have seen the effects of gene activation switching on protein noise. As in previous results also obtained in Dessalles et al. (2017), we find that in the limit where the gene is *off* most of the time, the negative feedback reduces noise at most by a factor of two. Additionally we find that noise can increase unboundedly for positive feedback.

Today, quasi-steady-state assumptions are frequently used when analyzing chemical reaction networks. While the intuition suggests the correct approach when approximating the system by a deterministic path, studying fluctuations is apparently much less obvious. In Kim et al. (2015), some special cases are studied when a straight-forward approximation of the fluctuations works. In our analysis, we use a new approach by Kang et al. (2014) and can also interpret all terms arising in (14), see Sect. 3.3.

Due to taking into account all potential sources of fluctuations the fit of simulations and theory (see e.g. Fig. 2) is excellent and improves on previous studies. There, noise arising from the gene switching its state has been averaged out, and only the recent approach of Kang et al. (2014) reveals the impact of these stochastic processes on the noise in protein numbers.

In their paper, Kang et al. (2014) gave as an example an approximation of noise for Michaelis–Menten kinetics and a model for virus infection. Their method relies mostly on solving a Poisson equation $L_2h=F_N-F$, where $L_2$ is the generator of the fast subsystem (gene and RNA in our example), $F_N$ and *F* describe the evolution of the slow system (protein) including all fluctuations and in the limit using the quasi-steady-state assumption, respectively. We stress that this approach is not only useful for equilibrium situations, but also for understanding noise if the slow system has not reached equilibrium yet, e.g. after a cell division.

It was argued that complexity of gene regulatory networks leads to a reduction in the level of noise, while certain network motifs always lead to increased levels of noise (Becskei and Serrano 2000; Cardelli et al. 2016). Experimentally, gene expression noise can be used to understand the dynamics of gene regulation (Munsky et al. 2012). Our analysis should provide an approach for distinguishing between different models of gene regulation based on measurements of noise levels.

## Recalling the approach of Kang et al. (2014)

We will consider a general system of chemical reactions with ${\mathcal {S}}$ as the set of chemical species and ${\mathcal {K}}$ the set of reactions; see also Anderson and Kurtz (2015) for further reference. The chemical reactions have the form

25

where $\nu _k = (\nu _{ks})_{s\in {\mathcal {S}}}$ and $\nu _k' = (\nu _{ks}')_{s\in {\mathcal {S}}}$ are vectors of chemical species, i.e. elements of ${\mathbb {N}}^{{\mathcal {S}}}$. For the dynamics, we assume mass action kinetics, i.e. we set

$$\begin{aligned} {\varLambda }_k(x)&= \lambda _k x^{\nu _k} := \lambda _k \prod _{s\in {\mathcal {S}}} x_s^{\nu _{ks}} \end{aligned}$$

for the reaction rate of reaction $k\in {\mathcal {K}}$. With

$$\begin{aligned} \zeta _k := \nu _k' - \nu _k, \end{aligned}$$

we can then define the dynamics of the Markov process $X = (X_s)_{s\in {\mathcal {S}}}$ through the process $R_k$, which describes the number of occurrences of reaction *k* up to time *t*. We have

$$\begin{aligned} R_k(t)&= Y_k\left( \int _0^t {\varLambda }_k(X(s))ds\right) \end{aligned}$$

for independent unit rate Poisson processes $Y_k, k\in {\mathcal {K}}$ and therefore

26 $$\begin{aligned} X(t) = X(0) + \sum _{k\in {\mathcal {K}}} \zeta _k R_k(t) = X(0) + \sum _{k\in {\mathcal {K}}} \zeta _k Y_k\left( \int _0^t {\varLambda }_k(X(s))ds\right) . \end{aligned}$$

We will tailor the results of Kang et al. (2014) to the special case we need in our gene expression example. This means that we can make use of several simplifications, e.g. on the form of the infinitesimal generator of the full process.

For some scaling parameter *N*, assume that $X = X^N = (X_{{\mathrm {s}}}^N, X_{{\mathrm {f}}}^N)$ is a Markov jump process with state space ${\mathbb {N}}^{d_{\text {s}}}\times {\mathbb {N}}^{d_{\text {f}}}$ such that for $V^N = (V_{{\mathrm {s}}}^N, V_{{\mathrm {f}}}^N)$ with $V_{{\mathrm {f}}}^N = X_{{\mathrm {f}}}^N$ and $V_{{\mathrm {s}}}^N = X_{{\mathrm {s}}}^N/N$, the system $V_{{\mathrm {s}}}^N$ is a slow (rescaled) sub-system and $V_{{\mathrm {f}}}^N$ is a fast sub-system. We assume that the generator $L^N$ of $X^N$ has the form

27 $$\begin{aligned} L^N = L^N_1 + NL_2^N, \end{aligned}$$

where $L^N_2$ describes the dynamics of $V^N_{{\mathrm {f}}}$, i.e. $L_2^Nf=0$ if *f* only depends on $v_{{\mathrm {s}}}$. Our goal is to show convergence

for some $V_{{\mathrm {s}}}$ and *U*. Therefore, we proceed as follows.

1. We have that (with the projection $\pi _{{\mathrm {s}}}$ on the slow species and $F^N := L^N\pi _{{\mathrm {s}}} = L_1^N\pi _{{\mathrm {s}}}$)
  $$\begin{aligned} M^{N}_1(t) := V_{{\mathrm {s}}}^N(t) - V_{{\mathrm {s}}}^N(0) - \int _0^t F^N\left( V_{{\mathrm {s}}}^N(s), V_{{\mathrm {f}}}^N(s)\right) ds \end{aligned}$$
  is a (local) martingale. For the convergence (28), we assume that
  for some unique process $V_{{\mathrm {s}}}$, which holds for where $\rho _N$ is the equilibrium of $V_{{\mathrm {f}}}^N$ for fixed slow species $v_s$. Thus, the convergence  holds with
  $$\begin{aligned} V_{{\mathrm {s}}}(t) = V_{{\mathrm {s}}}(0) + \int _0^t F\left( V_{{\mathrm {s}}}(s)\right) ds \end{aligned}$$
  and we have shown (28).
2. Note that
  $$\begin{aligned}&U^N(t) - U^N(0) \\&\quad = \sqrt{N} \left( V_{{\mathrm {s}}}^N(t) - V_{{\mathrm {s}}}(t)\right) - \sqrt{N}\left( V_{{\mathrm {s}}}^N(0) - V_{{\mathrm {s}}}(0)\right) \\&\quad = \sqrt{N}\left( M_1^{N}(t) + \int _0^t \left( F^N\left( V_{{\mathrm {s}}}^N(s), V_{{\mathrm {f}}}^N(s)\right) - F\left( V_{{\mathrm {s}}}^N(s)\right) \right) ds \right. \\&\qquad \left. + \int _0^t \left( F\left( V_{{\mathrm {s}}}^N\right) - F(V_{{\mathrm {s}}}) \right) ds\right) . \end{aligned}$$
  Assume that we (The ‘$\approx$’ is controlled by $\varepsilon _2^N$ below. Note that this is a Poisson equation.) With
  30 $$\begin{aligned} \begin{aligned} \varepsilon ^N_1(t)&:= \frac{1}{N} \left( h^N\left( V_{{\mathrm {s}}}^N(t), V_{{\mathrm {f}}}^N(t)\right) - h^N\left( V_{{\mathrm {s}}}^N(0), V_{{\mathrm {f}}}^N(0)\right) \right. \\&\quad \left. - \int _0^t L_1^N h^N\left( V_{{\mathrm {s}}}^N(s), V_{{\mathrm {f}}}^N(s)\right) ds\right) ,\\ \varepsilon _2^N(t)&:= - \int _0^t \left( L_2^Nh^N\left( V_{{\mathrm {s}}}^N(s), V_{{\mathrm {f}}}^N(s)\right) \right. \\&\quad \left. - (F^N\left( V_{{\mathrm {s}}}^N(s), V_{{\mathrm {f}}}^N(s)\right) - F\left( V_{{\mathrm {s}}}^N(s)\right) )\right) ds, \end{aligned} \end{aligned}$$
  we obtain that
  $$\begin{aligned} \begin{aligned} M^{N}_2(t)&:= \varepsilon _1^N(t) + \varepsilon _2^N(t) - \int _0^t \left( F^N\left( V_{{\mathrm {s}}}^N(s), V_{{\mathrm {f}}}^N(s)\right) - F\left( V_{{\mathrm {s}}}^N(s)\right) \right) ds \end{aligned} \end{aligned}$$
  is a (local) martingale. Hence
  $$\begin{aligned}&\sqrt{N} \left( V_{{\mathrm {s}}}^N(t) - V_{{\mathrm {s}}}(t)\right) - \sqrt{N}\left( V_{{\mathrm {s}}}^N(0) - V_{{\mathrm {s}}}(0)\right) \\&\quad = \sqrt{N}\left( M^{N,1}(t) - M^{N,2}(t) + \varepsilon _1^N(t) + \varepsilon _2^N(t) + \int _0^t \left( F\left( V_{{\mathrm {s}}}^N\right) - F(V_{{\mathrm {s}}}) \right) ds\right) . \end{aligned}$$
  We assume for $\varepsilon ^N_1, \varepsilon ^N_2$ from (30) that
3. In order to show convergence of $M^{N}_1 - M^{N}_2$, we use the Martingale Central Limit Theorem; see Appendix A.1 in Kang et al. (2014). Note that since the quadratic variation of all integrals $\int dt$ vanishes, we find that (recall the notation for Chemical Reaction Networks from (25)–(26), which we now equip with a superscript *N* to account for the scaling constant), with $z^{\otimes 2} = zz^\top$
  $$\begin{aligned} \begin{aligned} \left[ \sqrt{N}\left( M^N_1 - M^N_2\right) \right] _t&= N\left[ V^N_{{\mathrm {s}}} - \frac{1}{N}\left( h^N\left( V^N_{{\mathrm {s}}}, V^N_{{\mathrm {f}}}\right) \right) \right] _t \\&= \frac{1}{N} \sum _{k \in {\mathcal {K}}} \int _0^t \left( {\zeta }_{k{\mathrm {s}}} + h^N\left( V^N_{{\mathrm {s}}}(s-), V^N_{{\mathrm {f}}}(s-)\right) \right. \\&\quad \left. - h^N\left( V^N_{{\mathrm {s}}}(s-) + \frac{1}{N} \zeta _{k{\mathrm {s}}}, V^N_{{\mathrm {f}}}(s-) +\zeta _{k{\mathrm {f}}}\right) \right) ^{\otimes 2} dR_k^N(s), \end{aligned} \end{aligned}$$
  where $\zeta _{k{\mathrm {s}}}$ and $\zeta _{k{\mathrm {f}}}$ are the stochiometric changes of the *k*th reaction in the slow and fast subsystem, respectively. Note that in all applications, we will have that $R_k^N$ either changes slowly, or changes fast and can thus be approximated by a deterministic curve, such that
  $$\begin{aligned} \begin{aligned} \lim _{N\rightarrow \infty }[\sqrt{N}(M^N_1 - M^N_2)]_t&= \lim _{N\rightarrow \infty } \frac{1}{N} \sum _{k \in {\mathcal {K}}} \int _0^t \left( {\zeta }_{k{\mathrm {s}}} + h^N\left( V^N_{{\mathrm {s}}}(s), V^N_{{\mathrm {f}}}(s-)\right) \right. \\&\quad \left. - h^N\left( V^N_{{\mathrm {s}}}(s) + \frac{1}{N} \zeta _{k{\mathrm {s}}}, V^N_{{\mathrm {f}}}(s-) +\zeta _{k{\mathrm {f}}}\right) \right) ^{\otimes 2} {\varLambda }^N_k(s) ds. \end{aligned} \end{aligned}$$
  Now, for the equilibrium $\rho _N$ of the fast species for given concentration of slow species, $v_{{\mathrm {s}}}$, if we have that
  where the right hand side is a deterministic, absolutely continuous, ${\mathbb {R}}^{{\mathrm {s}} \times {\mathrm {s}}}$-valued function with non-negative time-derivative. Hence we know from the martingale central limit theorem (see Appendix A.1 in Kang et al. 2014) that
  where *M* satisfies
  $$\begin{aligned} dM&= \sqrt{c(V(t))} dW. \end{aligned}$$
4. Concluding, if $F\in {\mathcal {C}}^1({\mathbb {R}}^{d_{{\mathrm {s}}}})$ with
  $$\begin{aligned} F\left( V^N_{{\mathrm {s}}}\right) - F(V_{{\mathrm {s}}}) = \frac{1}{\sqrt{N}} \left( \nabla F(V_{{\mathrm {s}}}) U^N+ o(1)\right) \end{aligned}$$
  we find that, if , then
  $$\begin{aligned} U(t) - U(0) = \int _0^t \nabla F(V_{{\mathrm {s}}}(r)) U_r dr + \int _0^t \sqrt{c(V(r))} dW. \end{aligned}$$
  This gives (29).

## Proof of Theorem 2

### Proof

We will make use of notation from Appendix A and have to show (➊)–(➍) from Appendix A in all cases. Note that the function *F* from Theorem 1 already satisfies (➊). In all cases, the system $(V_{\text {on}}, V_R, V_P)$ is a Markov process with a generator of the form (27) with

$$\begin{aligned} L_1^N f(u,r,v_P)&= \kappa _3 r N \left( f \left( u,r,v_P+\frac{1}{N}\right) - f(u,r,v_P)\right) \\&\quad + v_P\nu _3 N\left( f\left( u,r,v_P-\frac{1}{N}\right) - f(u,r,v_P)\right) \\&= (\kappa _3 r - v_p\nu _3)\frac{\partial f}{\partial v_P}(u,r,v_P) + o(1) \end{aligned}$$

(and different operators $L_2^N$). This already implies that for all cases

$$\begin{aligned} F^N(r,v_P) = \kappa _3 r - \nu _3 v_P. \end{aligned}$$

For ,

$$\begin{aligned} L_2^Nf(u,r,v_P)&= (M-u)\kappa _1^+ (f(u+1,r,v_P) - f(u,r,v_P)) \\&\quad + u \kappa _1^-(f(u-1,r,v_P) - f(u,r,v_P)) \\&\quad + \kappa _2 u (f(u,r+1,v_P) - f(u,r,v_P))\\&\quad + \nu _2 r (f(u,r-1,v_P) - f(u,r,v_P)). \end{aligned}$$

From (➋) and (4), we see that we need to solve

$$\begin{aligned} L^N_2 h^N&= \kappa _3 r - \frac{M\kappa _1^+\kappa _2\kappa _3}{\nu _2\left( \kappa _1^- + \kappa _1^+\right) }. \end{aligned}$$

Choosing the Ansatz

$$\begin{aligned} h^N(u,r,v_P) = ug_1(v_P) + rg_2(v_P), \end{aligned}$$

we obtain the following equation which we need to solve

$$\begin{aligned} (M \kappa _1^+ - u(\kappa _1^+ +\kappa _1^-)) g_1(v_P) + (\kappa _2 u - \nu _2 r) g_2(v_P) {\mathop {=}\limits ^{!}} \kappa _3 r - \frac{M\kappa _1^+\kappa _2\kappa _3}{\nu _2\left( \kappa _1^- + \kappa _1^+\right) }. \end{aligned}$$

Solving for $g_2$ and then for $g_1$, we obtain

$$\begin{aligned} g_2(v_P)&= -\frac{\kappa _3}{\nu _2}, \qquad g_1(v_P) = -\frac{\kappa _2 \kappa _3}{\nu _2 \left( \kappa _1^- + \kappa _1^+\right) }. \end{aligned}$$

Then,  since $h^N$ is bounded in *N* and $\varepsilon _2^N=0$ by construction. Hence, we have shown (➌). For (➍), if $\pi$ is the equilibrium of the fast species *U*, *R* for given value $v_P$ of the slow species as in Theorem 1, we have that

$$\begin{aligned}&\frac{1}{N} \sum _{k=1}^6 {\mathbb {E}}_\pi \left[ \left( \zeta _{kP} + h^N(U, R,v_P) - h^N\left( U + \zeta _{k\text {on}}, R + \zeta _{kr}, v_P + \frac{1}{N} \zeta _{kP}\right) \right) ^2 {\varLambda }_k(v_P)\right] \\&\quad = 2(g_1(v_P))^2 M \frac{\kappa _1^-\kappa _1^+}{\kappa _1^- + \kappa _1^+} + (g_2(v_P))^2 \left( \frac{M\kappa _1^+ \kappa _2}{\kappa _1^- + \kappa _1^+} + \frac{M\kappa _1^+ \kappa _2}{\nu _2\left( \kappa _1^- + \kappa _1^+\right) } \nu _2\right) \\&\qquad + \left( \frac{M \kappa _1^+ \kappa _2 \kappa _3}{\nu _2\left( \kappa _1^- + \kappa _1^+\right) } + \nu _3 v_P\right) \\&\quad = \frac{M\kappa _1^+}{\kappa _1^- + \kappa _1^+} \left( \frac{2 \kappa _1^- \kappa _2^2 \kappa _3^2}{\nu _2^2(\kappa _1^- + \kappa _2^+)^2} + \frac{2\kappa _2 \kappa _3^2}{\nu _2^2} + \frac{\kappa _2 \kappa _3}{\nu _2}\right) + \nu _3 v_P. \end{aligned}$$

For , all calculations above are the same, but with $\kappa _1^-$ replaced by $\kappa _1^\ominus v_P$, and for , all calculations are the same with $\kappa _1^+$ replaced by $\kappa _1^\oplus v_P$. $\square$

## The two-dimensional Ornstein–Uhlenbeck process

We recall results for the two-dimensional Ornstein–Uhlenbeck process; see e.g. Gardiner (2009). They are needed when refining the Fano factor in Appendix D.

### Theorem 3

(Stationary variance of two-dimensional Ornstein–Uhlenbeck process) Let $X=(X_1,X_2)$ solve

$$\begin{aligned} dX = -A X dt + B dW, \end{aligned}$$

where $A, B \in {\mathbb {R}}^{2\times 2}$. Then, if all eigenvalues of *A* have positive real part, the stationary solution $X_0$ of the SDE has ${\mathbb {E}}[X_0]=0$ and

$$\begin{aligned} {\mathbf {E}}[X_0 X_0^\top ] = \frac{(A-(\mathrm{tr}A)E_2)BB^\top (A^\top - (\mathrm{tr}A)E_2) + (\det A)BB^\top }{2(\det A)(\mathrm{tr}A)}. \end{aligned}$$

### Proof

Using partial integration, it is easy to see that this SDE is solved by

$$\begin{aligned} X_t = e^{-At} X_0 + \int _0^t e^{-A(t-s)} B dW. \end{aligned}$$

If all eigenvalues of *A* have positive real part, the stationary solution of the SDE has the distribution

$$\begin{aligned} X_0 = \int _{-\infty }^0 e^{As} B dW_s. \end{aligned}$$

In particular, ${\mathbb {E}}[X_0]=0$ and

$$\begin{aligned} {\mathbb {E}}[X_0 X_0^\top ]&= \int _{-\infty }^0 e^{As}B B^\top e^{A^\top s}ds. \end{aligned}$$

In order to compute the right hand side, we note that, for any $2\times 2$-matrix $$A = \left( \begin{matrix} a &{}\quad b \\ c &{}\quad d\end{matrix}\right) $$, we have that (for the unit matrix $I_2$)

$$\begin{aligned} A^2&= \left( \begin{matrix} a^2 + bc &{}\quad ab + bd \\ ca + dc &{}\quad cb + d^2\end{matrix}\right) = \left( \begin{matrix} (a+d)a - (ad-bc) &{}\quad (a+d)b \\ (a+d)c &{}\quad (a+d)d - (ad-bc)\end{matrix}\right) \\&=(\mathrm{tr}A)A - (\det A)I_2. \end{aligned}$$

Hence, we can write

$$\begin{aligned} e^{As} = \alpha _s + \beta _s A, \quad e^{A^\top s} = \alpha _s + \beta _s A^\top , \end{aligned}$$

i.e.

31 $$\begin{aligned} {\mathbb {E}}[X_0 X_0^\top ]= & {} \int _{-\infty }^0 (\alpha _s + \beta _s A) B B^\top (\alpha _s + \beta _s A^\top )ds \nonumber \\= & {} \alpha B B^\top + \beta (ABB^\top + BB^\top A^\top ) + \gamma ABB^\top A^\top \nonumber \\= & {} \gamma \left( A + \frac{\beta }{\gamma }\right) (BB^\top ) \left( A^\top + \frac{\beta }{\gamma }\right) + \left( \alpha - \frac{\beta ^2}{\gamma }\right) BB^\top \end{aligned}$$

for

$$\begin{aligned} \alpha = \int _{-\infty }^0 \alpha _s^2 ds, \quad \beta = \int _{-\infty }^0 \alpha _s \beta _s ds, \quad \gamma = \int _{-\infty }^0 \beta _s^2 ds. \end{aligned}$$

We then write

$$\begin{aligned} BB^\top&= \int _{-\infty }^0 \frac{d}{ds} e^{As}B B^\top e^{A^\top s} ds = A{\mathbb {E}}[X_0 X_0^\top ] + {\mathbb {E}}[X_0 X_0^\top ]A^\top \\&= \alpha (ABB^\top + BB^\top A) + 2\beta ABB^\top A^\top + \beta (A^2 BB^\top + BB^\top (A^\top )^2) \\&\quad + \gamma (A^2 BB^\top A^\top + ABB^\top (A^\top )^2) \\&= (\alpha - \gamma (\det A))(ABB^\top + BB^\top A) + 2(\beta + \gamma (\mathrm{tr}A)) ABB^\top A^\top \\&\quad + \beta (\mathrm{tr}A) (A BB^\top + BB^\top A^\top )- 2\beta (\det A) BB^\top \\&= - 2\beta (\det A) BB^\top + 2(\beta + \gamma (\mathrm{tr}A)) ABB^\top A^\top \\&\quad + (\alpha + \beta (\mathrm{tr}A) - \gamma (\det A))(ABB^\top + BB^\top A), \end{aligned}$$

which is only possible if

$$\begin{aligned} 1 + 2\beta (\det A)&= 0, \\ \beta + \gamma (\mathrm{tr}A)&= 0, \\ \alpha + \beta (\mathrm{tr}A) - \gamma (\det A)&= 0, \end{aligned}$$

i.e.

$$\begin{aligned} \alpha = \frac{\det A +(\mathrm{tr}A)^2}{2(\det A)(\mathrm{tr}A)}, \qquad \beta = -\frac{1}{2\det A}, \qquad \gamma = \frac{1}{2(\det A)(\mathrm{tr}A)}. \end{aligned}$$

Combining this with (31) then gives the result. $\square$

### Corollary 1

(Diagonal matrix *B*) Note that if

then

$$\begin{aligned} {\mathbb {E}}[X_0 X_0^\top ] = \frac{ \left( \begin{matrix} \rho b^2 + \lambda (d \cdot \mathrm{tr}(A) - bc) &{}\quad - (\rho ab + \lambda cd) \\ - (\rho ab + \lambda cd) &{}\quad \rho (a \cdot \mathrm{tr}(A) - bc) + \lambda c^2\end{matrix}\right) }{2(ad-bc)(a+d)}. \end{aligned}$$

### Proof

Indeed, $$A - \mathrm{tr}(A)E_2 = \left( \begin{matrix} -d &{} \quad b \\ c &{}\quad -a\end{matrix}\right) $$, so

$$\begin{aligned} (A-(\mathrm{tr}A)E_2)BB^\top (A^\top - (\mathrm{tr}A)E_2)&= \left( \begin{matrix} -\lambda d &{}\quad \rho b \\ \lambda c &{}\quad -\rho a\end{matrix}\right) \left( \begin{matrix} -d &{}\quad c \\ b &{}\quad -a\end{matrix}\right) \\&= \left( \begin{matrix} \rho b^2 + \lambda d^2 &{}\quad - (\rho ab + \lambda cd) \\ - (\rho ab + \lambda cd) &{}\quad \rho a^2 + \lambda c^2 \end{matrix}\right) . \end{aligned}$$

$$\square $$

## Refining the Fano factor

Here, we study the case

$$\begin{aligned} \lambda _3 = {\widetilde{\kappa }}_3, \qquad \mu _2 = {\widetilde{\nu }}_2 \end{aligned}$$

which leads to $X_R = O(N)$, such that we have the scaling $\alpha _{\text {off}} = \alpha _{\text {on}}=0$ and $\alpha _R=\alpha _P=1$; compare with (1). In particular, we use that—see (2)—$V_R = X_R/N, V_P = X_P/N$. Here, $V_{\text {on}} = X_{\text {on}}$ is fast and $(V_R, V_P)$ are slow. Note that in this case, we have that $(V_R^N, V_P^N)\Rightarrow (V_R, V_P)$ with

We will denote the unique solution of $F(V_R, V_P)=0$ by $(v_R^*, v_P^*)$.

### Theorem 4

(Central Limit Theorem) Let $V_R^N, V_R, V_P^N, V_P$ and *F* be as above and . Then, for the models  and , , where $(U_R, U_P)$ solves

32 $$\begin{aligned} U_R(t)&= U_R(0) + \int _0^t \sqrt{c_R(V_P(s))}dW(s) \nonumber \\&\quad - \int _0^t \left( {\widetilde{\nu }}_2 U_R(s) + d_R(V_P(s))U_P(s)\right) ds, \nonumber \\ U_P(t)&= U_P(0) + \int _0^t \sqrt{{\widetilde{\kappa }}_3 V_R(s) + \nu _3 V_P(s)}dW'(s) \nonumber \\&\quad + \int _0^t \left( {\widetilde{\kappa }}_3 U_R(s) - \nu _3 V_P(s) U_P(s)\right) ds \end{aligned}$$

with $W, W'$ independent Brownian motions,

33

and

34

### Remark 2

In equilibrium, we have

35

### Proof

First, note that

Again, we have to show (➊)–➍) from Appendix A in all cases for *F* as above, which already satisfies (➊). We focus on  first. The system $(V_{\text {on}}, V_R, V_P)$ is a Markov process with a generator of the form (27) with

$$\begin{aligned} L_1^N f(u,v_R,v_P)&= (\kappa _2 u - v_R{\widetilde{\nu }}_2)\frac{\partial f}{\partial v_R}(u,v_R,v_P) \\&\quad + ({\widetilde{\kappa }}_3 v_R - v_P\nu _3)\frac{\partial f}{\partial v_P}(u,v_R,v_P) + o(1), \\ L_2^N f(u,v_R,v_P)&= (M-u) \kappa _1^+(f(u+1, v_R, v_P) - f(u, v_R, v_P)) \\&\quad + u \kappa _1^- (f(u-1, v_R, v_P) - f(u, v_R, v_P)). \end{aligned}$$

This implies that

$$\begin{aligned} F^N(u,v_R,v_P) = \left( \begin{matrix} \kappa _2 u - {\widetilde{\nu }}_2 v_R \\ {\widetilde{\kappa }}_3 v_R - \nu _3 v_P\end{matrix}\right) . \end{aligned}$$

Hence, for (➋), we have to solve

$$\begin{aligned} L_2^Nh^N(u, v_R, v_P)&= \left( \begin{matrix} \displaystyle \kappa _2 u - \frac{M\kappa _1^+\kappa _2}{\kappa _1^- + \kappa _1^+} \\ 0 \end{matrix}\right) , \end{aligned}$$

which is

$$\begin{aligned} h^N(u, v_R, v_P)&= \left( \begin{matrix} -u \displaystyle \frac{\kappa _2}{\kappa _1^- + \kappa _1^+} \\ 0 \end{matrix}\right) . \end{aligned}$$

For the quadratic variation in (➍), we find that with $z^{\otimes 2} = zz^\top$ This shows the assertion for . The cases  and  are similar, if we change $\kappa _1^-$ by $\kappa _1^\ominus v_P$ for  and $\kappa _1^+$ by $\kappa _1^\oplus v_P$ for . $\square$

### Equilibrium Fano factor$\ldots$

Let us start in equilibrium, i.e. $V_R(0)=v_R^*, V_P(0)=v_P^*$. Then, we will plug in $c_R$ from (35), and obtain Ornstein–Uhlenbeck processes in all cases. Since they are two-dimensional, their equilibrium (normal) distribution can be computed (see Appendix C).

#### $\ldots$for

We obtain Hence, with $U=(U_R, U_P)^\top$, in equilibrium, from Corollary 1,

36

Therefore,

37

#### $\ldots$for

Here, we obtain Hence, in equilibrium, and if $\cdots$ denote the quantities from (36), and for $b = \frac{\kappa _1^\ominus v_P^*{\widetilde{\nu }}_2 \nu _3 }{\left( \kappa _1^\ominus v_P^*+ \kappa _1^+\right) {\widetilde{\kappa }}_3}$,

38

So,

39

#### $\ldots$for

We obtain In equilibrium, we now have exactly (38) but with $b = - \frac{\kappa _1^- {\widetilde{\nu }}_2\nu _3}{\left( \kappa _1^- + \kappa _1^\oplus v_P^*\right) {\widetilde{\kappa }}_3}$. Hence,

40

## Results in unscaled parameter notation

In this section we state the formulas from Sect. 3 in terms of the unscaled parameters, i.e. $\lambda$’s and $\mu$’s. The deterministic approximation or law of large numbers for the number of protein is given in the following remark (compare with Theorem 1). Throughout, we have $X_P \approx NV_P$.

### Remark 3

(*Law of large numbers in unscaled parameters*) Given the assumptions of Theorem 1 we find that approximately (see (4))

with equilibria (see (5))

Next, we give the unscaled version of the central limit theorem derived in the main text (see also Theorem 2).

### Remark 4

(*Central limit theorem in unscaled parameters*) Assuming the same setting as in Theorem 2 and writing down the resulting formula (7) in unscaled parameters we find

41 $$\begin{aligned} dX_P&= Ndv_P + \sqrt{N}dU \approx \left( NF(v_P) + \sqrt{N}F'(v_P)U\right) dt + \sqrt{{\widetilde{b}}(X_P)} dW \end{aligned}$$

with

Using Theorem 2 we can now write down the Fano factor obtained in equilibrium:

### Remark 5

(*Fano factor in unscaled parameters*) From (41), we find that, with $X_P\left( 0\right) = X_P^*$, in equilibrium (see (14))

42

The comparison of the fluctuations in neutral version negative and positive feedback reads for unscaled parameters (see (16))

43

For completeness, we also give the unscaled version of the refined Fano factor derived in Sect. 3.6 and Appendix D. From (18),

44

with

## References

[CR1] Anderson D, Kurtz TG (2015). Stochastic analysis of biochemical systems.

[CR2] Balázsi G, van Oudenaarden A, Collins J (2011). Cellular decision making and biological noise: from microbes to mammals. Cell.

[CR3] Ball K, Kurtz TG, Popovic L, Rempala G (2006). Asymptotic analysis of multiscale approximations to reaction networks. Ann Appl Probab.

[CR4] Bar-Even A, Paulsson J, Maheshri N, Carmi M, O’Shea E, Pilpel Y, Barkai N (2006). Noise in protein expression scales with natural protein abundance. Nat Genet.

[CR5] Becskei A, Serrano L (2000). Engineering stability in gene networks by autoregulation. Nature.

[CR6] Bokes P, King JR, Wood A, Loose M (2012). Multiscale stochastic modelling of gene expression. J Math Biol.

[CR7] Cardelli L, Csikász-Nagy A, Dalchau N, Tribastone M, Tschaikowski M (2016). Noise reduction in complex biological switches. Sci Rep.

[CR8] Darling RWR (2002) Fluid limits of pure jump Markov processes: a practical guide, 1–16. arxiv preprint arXiv:math/0210109

[CR9] Dessalles R, Fromion V, Robert P (2017). A stochastic analysis of autoregulation of gene expression. J Math Biol.

[CR10] Eldar A, Elowitz MB (2010). Functional roles for noise in genetic circuits. Nature.

[CR11] Elowitz MB, Levine AJ, Siggia ED, Swain PS (2002). Stochastic gene expression in a single cell. Science.

[CR12] Ethier SN, Kurtz TG (1986). Markov processes: characterization and convergence. Wiley series in probability and mathematical statistics.

[CR13] Fraser D, Kærn M (2009). A chance at survival: gene expression noise and phenotypic diversification strategies. Mol Microbiol.

[CR14] Gardiner C (2009). Stochastic methods. A handbook for the natural and social sciences.

[CR15] Gillespie D (1977). Exact stochastic simulation of coupled chemical reactions. J Phys Chem.

[CR16] Grönlund A, Lötstedt P, Elf J (2013). Transcription factor binding kinetics constrain noise suppression via negative feedback. Nat Commun.

[CR17] Hornos JEM, Schultz D, Innocentini G, Wang J, Walczak A, Onuchic J, Wolynes PG (2005). Self-regulating gene: an exact solution. Phys Rev E.

[CR18] Hornung G, Barkai N (2008). Noise propagation and signaling sensitivity in biological networks: a role for positive feedback. PLoS Comput Biol.

[CR19] Kaern M, Elston TC, Blake WJ, Collins JJ (2005). Stochasticity in gene expression: from theories to phenotypes. Nat Rev Genet.

[CR20] Kang HW, Kurtz T, Popovic L (2014). Central limit theorems and diffusion approximations for multiscale Markov chain models. Ann Appl Probab.

[CR21] Kim JK, Josić K, Bennett MR (2015). The relationship between stochastic and deterministic quasi-steady state approximations. BMC Syst Biol.

[CR22] Kuehn C (2015). Multiple time scale dynamics.

[CR23] Kumar N, Singh A, Kulkarni RV (2015). Transcriptional bursting in gene expression: analytical results for general stochastic models. PLoS Comput Biol.

[CR24] Kurtz T (1970). Limit theorems for sequences of jump Markov processes approximating ordinary differential processes. J Appl Probab.

[CR25] Kurtz T (1970). Solutions of ordinary differential equations as limits of pure jump Markov processes. J Appl Probab.

[CR26] Lestas I, Vinnicombe G, Paulsson J (2010). Fundamental limits on the suppression of molecular fluctuations. Nature.

[CR27] Li GW, Xie XS (2011). Central dogma at the single-molecule level in living cells. Nature.

[CR28] Maamar H, Raj A, Dubnau D (2007). Noise in gene expression determines cell fate in bacillus subtilis. Science.

[CR29] Mitosch K, Rieckh G, Bollenbach T (2017). Noisy response to antibiotic stress predicts subsequent single-cell survival in an acidic environment. Cell Syst.

[CR30] Munsky B, Neuert G, van Oudenaarden A (2012). Using gene expression noise to understand gene regulation. Science.

[CR31] Pardoux E, Veretennikov AY (2001). On Poisson equation and diffusion approximation 1. Ann Probab.

[CR32] Pardoux E, Veretennikov AY (2003). On Poisson equation and diffusion approximation 2. Ann Probab.

[CR33] Paulsson J (2005). Models of stochastic gene expression. Phys Life Rev.

[CR34] Raj A, van Oudenaarden A (2008). Nature, nurture, or chance: stochastic gene expression and its consequences. Cell.

[CR35] Ramos A, Innocentini G, Hornos J (2011). Exact time-dependent solutions for a self-regulating gene. Phys Rev E.

[CR36] Ramos A, Hornos J, Reinitz J (2015). Gene regulation and noise reduction by coupling of stochastic processes. Phys Rev E.

[CR37] Raser JM, O’Shea EK (2005). Noise in gene expression: origins, consequences, and control. Science.

[CR38] Seegel LA, Slemrod M (1989). The quasi-steady-state assumption: a case study in perturbation. SIAM Rev.

[CR39] Shahrezaei V, Swain PS (2008). Analytical distributions for stochastic gene expression. Proc Natl Acad Sci USA.

[CR40] Silva-Rocha R, de Lorenzo V (2010). Noise and robustness in prokaryotic regulatory networks. Annu Rev Microbiol.

[CR41] Singh A (2014). Transient changes in intercellular protein variability identify sources of noise in gene expression. Biophys J.

[CR42] Singh A, Soltani M (2013). Quantifying intrinsic and extrinsic variability in stochastic gene expression models. PLoS ONE.

[CR43] Swain PS (2004). Efficient attenuation of stochasticity in gene expression through post-transcriptional control. J Mol Biol.

[CR44] Swain PS, Elowitz MB, Siggia ED (2002). Intrinsic and extrinsic contributions to stochasticity in gene expression. Proc Natl Acad Sci USA.

[CR45] Thattai M, van Oudenaarden A (2001). Intrinsic noise in gene regulatory networks. Proc Natl Acad Sci.

[CR46] Wang Z, Zhang J (2011). Impact of gene expression noise on organismal fitness and the efficacy of natural selection. Proc Natl Acad Sci.

